# Changes in higher order cognitive function between four watch keeping schedules

**DOI:** 10.1093/sleepadvances/zpae044

**Published:** 2024-07-05

**Authors:** Jacob R Guzzetti, Isabella Marando, Raymond W Matthews, Mikaela S Owen, Crystal Yates, Siobhan Banks

**Affiliations:** Sleep and Chronobiology Laboratory, Behaviour-Brain-Body Research Centre, University of South Australia, Adelaide, SA, Australia; Sleep and Chronobiology Laboratory, Behaviour-Brain-Body Research Centre, University of South Australia, Adelaide, SA, Australia; Appleton Institute, Central Queensland University, Wayville, SA, Australia; Sleep and Chronobiology Laboratory, Behaviour-Brain-Body Research Centre, University of South Australia, Adelaide, SA, Australia; Sleep and Chronobiology Laboratory, Behaviour-Brain-Body Research Centre, University of South Australia, Adelaide, SA, Australia; Sleep and Chronobiology Laboratory, Behaviour-Brain-Body Research Centre, University of South Australia, Adelaide, SA, Australia

**Keywords:** shift work, cognitive function, sleep restriction, neurobehavioral performance

## Abstract

Maritime industries utilize many different watch keeping schedules to maintain vigilance and crew safety around the clock. These schedules can be fatiguing, negatively impacting vigilant attention. This has led to the consideration of schedules that might allow for more sleep time, but how these schedules impact higher order cognitive function remains unclear. These schedules require assessment with tasks that are relevant to real-world operations on maritime vessels. This study investigated the effect of four schedules on higher order cognitive function. *N* = 27 (16 female) participants were recruited to a 10-day laboratory study, comparing four schedules. The schedules investigated were eight-on/eight-off/four-on/four-off (8/8/4/4) with sleep from 09:30 to 16:00 (condition A); six-on/six-off (6/6) with sleep from 08:30 to 12:30 and 21:30 to 00:00 (condition B); four-on/four-off (4/4/4/4/4/4) with sleep from 18:00 to 00:30 (condition C); and four-on/four-off (4/4/4/4/4/4) with sleep from 01:30 to 08:00 (condition D). Higher order cognitive function was assessed 2–3× daily whilst “on watch” using tests of visual scanning, learning, working memory, mental flexibility, and visuomotor control. Conditions were ranked and stability of performance on watch was compared between conditions using Kruskal–Wallis tests. Cognitive function within condition B was ranked the worst for most of the tasks. However, the stability of higher order cognitive function was poorest across the waking day within condition A. These findings highlight the variability in cognitive capacities during different watch keeping schedules.

**Statement of Significance**

Demanding shiftwork schedules which require long workdays and little time for rest are required in continuous operational settings. Overtime, this can lead to fatiguing conditions for workers, and declining cognitive performance which can result in costly errors or death. There is longstanding interest in the continuous development of shiftwork schedules which can adapt to ever-evolving operational requirements. Studies have largely relied upon basic tests of reaction time to examine cognitive performance across different schedules and infer feasibility to the real-world. This work addresses the critical need for the deeper evaluation of cognitive performance and offers valuable insight into schedules that are currently in use and may be used in the future.

Watch keeping is a variable and demanding type of shiftwork frequently used in industrial operational settings in which around-the-clock vigilance is vital. Typically, watch keeping schedules list durations of both “on-” and “off-watch” periods (e.g. “4h-on/8h-off,” or just “4/8,” denoting a 4-hour watch keeping shift, followed by 8 hours off, in repetition). In addition to fulfilling other duties, watch keepers working in teams or sections maintain either daily “fixed” (or “stable” or “standing”) watch timing for the duration of an operational period or are “rotating” through various sections of a schedule which can be of 24 hours or non-24 hours structure (e.g. a 3-section 6/12 schedule). Thus, incompatibility with a 24-hour day, inconsistent activity timing, work at night, and multiple shifts per 24 hours characterize watch keeping schedules. Impaired cognitive performance has been reported in watch keepers underway on submarines and surface ships [1, 2].

In one of the earliest field reports of submarine 4/8 watch keeping conditions, Kleitman advocated for the importance of exploring alternative schedules in both the field and under controlled laboratory conditions and called for the introduction of cognitive performance measures for comparison [3]. Since then, the inclusion of basic cognitive performance measures has assisted in identifying preferable watch keeping schedules in both the laboratory and the field. Reaction time (RT) or vigilance (PVT; psychomotor vigilance test) assessments have been used to explore cognitive performance in studies comparing watch keeping schedules. Kongsvik and Størkersen reported vigilance on a Norwegian supply vessel under the 2-section 8/8/4/4 to be no different to the 2-section 6/6, the most common schedule across maritime operations [4]. However, van Leeuwen et al. reported better vigilance under the 4/8 than the 6/6 in a 6-day laboratory study of real bridge officers [5]. Similarly, better vigilance has been reported under the 4-section 3/9 schedule than the modified 4-section rotating 6/18 on the USN Arleigh Burke, the 3-section 5/10 on the USS NIMITZ, the 4-section 5/15, and the 6/6, both on the USS JASON DUNHAM [6–9]. Skornyakov and colleagues reported vigilance during the 3/9 to also be preferable to the 5/15 in a 6-day laboratory study [10]. Twenty-four hours structure in conjunction with several teams/sections and short watch lengths may confer high feasibility to the 3/9 schedule. Overall, these studies highlight that different watch keeping schedules can impact upon the vigilance of the watch keepers; however, the operational demands of watch keepers often require higher order cognitive function which cannot be assessed with vigilance and simple RT tasks. Thus, Kleitman’s call for the introduction of performance measures has been echoed by many authors [11–13].

Tests of higher order cognitive function, which demand holding and working with information in the mind over time, have been used in watch keeping field studies but are rarely compared between schedules. An assessment of working memory and sustained attention, respectively with the N-back task and the sustained attention to response task, carried out in the field by Myllylä et al. yielded no differences between two 2-section schedules: a rotating 4/4 and a 4/4/2/2/6/6 [14]. Alternatively, laboratory watch keeping studies are more conducive to comparing higher order cognition because they are not impacted by the distractions and interruptions that are inherent in real-world testing. Accordingly, Miller et al. examined visual-spatial processing, simple motor speed, basic computational skills, abstract reasoning, and attention in a series of 6-day laboratory studies of 6/12, 4/8, and 6/6/6/12/6/6/6/24 schedules [15]. They found that the only change was in a simple Running Memory task which was at its worst during the 6/12 schedule. To date, no studies comparing alternatives to the popular 6/6 schedule have examined higher order cognitive function in the laboratory beyond selective attention using Stroop task, which, along with vigilance, was found to be similar to the 4/8 [16].

Some of the most beneficial schedules that have been identified with the aid of performance measures, rely on sufficient personnel to support up to four watch sections (e.g. the 3/9 and the 6/18) [2, 9, 10].Vessel size, mission purpose and duration, and budgetary and manning capabilities vary broadly among vigilant operations and dictate the number of watch sections available and ultimately the schedules that can be adopted by commanding officers. In addition to the watch sections available, these factors may also impose a limit to the maximum duration of a watch permissible for an operation. If a watch keeping schedule is supported by only two sections, such as the 6/6, limiting the maximum watch duration to 6 hours also means limiting the maximum off-watch duration to 6 hours. Such schedules prohibit obtaining adequate consolidated daily sleep during one off-watch period; thus, sleep is often split between multiple off-watch periods in the same day [17]. Furthermore, splitting sleep often requires sleep to be attempted in the daytime when initiating and maintaining sleep is difficult due to the low circadian propensity. This can make it challenging to meet sleep requirements with the off time provided, and on long maritime voyages, this can lead to chronic sleep restriction and fatigue, both of which are known to negatively affect performance. Therefore, investigation into watch keeping schedules which could also minimize the degradation of performance has persisted.

Cognitive performance modeling derived from activity timing by Paul et al. suggested cognitive effectiveness during the 8/8/4/4 and 4/4/4/4/4/4 (“straight fours”) schedules are different to the 6/6 [18]. However, comparing cognitive performance between these schedules has yet to be done. The present study aimed to examine higher order cognitive function important to watch keeping including learning and mental flexibility between four watch keeping schedule sections from 2 and 3-section watch schedules.

## Methods

### Participants

**Figure 1.**
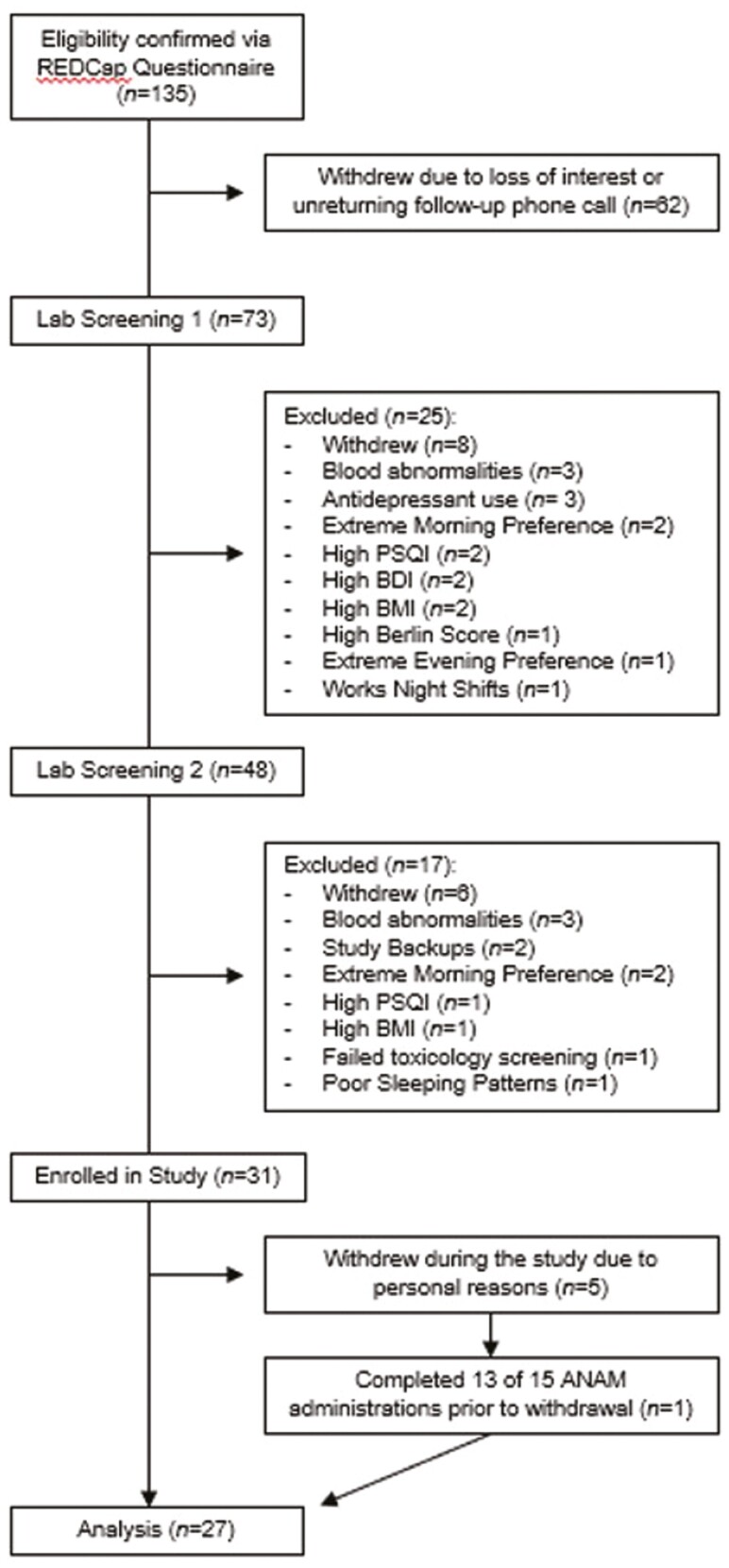
Participant CONSORT diagram. Participant CONSORT diagram for the study shows that 31 participants were originally enrolled in this study. Five withdrew mid-study. Data were included for one of the five participants that withdrew, as they completed most testing sessions.

Participants were recruited through paper and electronic flyers advertised on the university campus and social media platforms (e.g. Facebook and Twitter), respectively. Respondents were prompted to complete an initial REDCap screening questionnaire, for which items largely pertained to psychological and physiological health, sleep, and drug and alcohol use. Those with sleep disorders, poor sleep as determined by the Pittsburgh Sleep Quality Index, or scores outside of the *intermediate* range on the Composite Scale of Morningness were excluded from the study (Figure 1) [19, 20]. Extreme caffeine, regular alcohol (both >2 drinks per day), or drug use was also exclusionary, and transmeridian travel was proscribed for three months prior to study. All female participants were in the follicular phase of their menstrual cycle at the time of their study run.

### Laboratory setting and procedure

**Figure 2.**
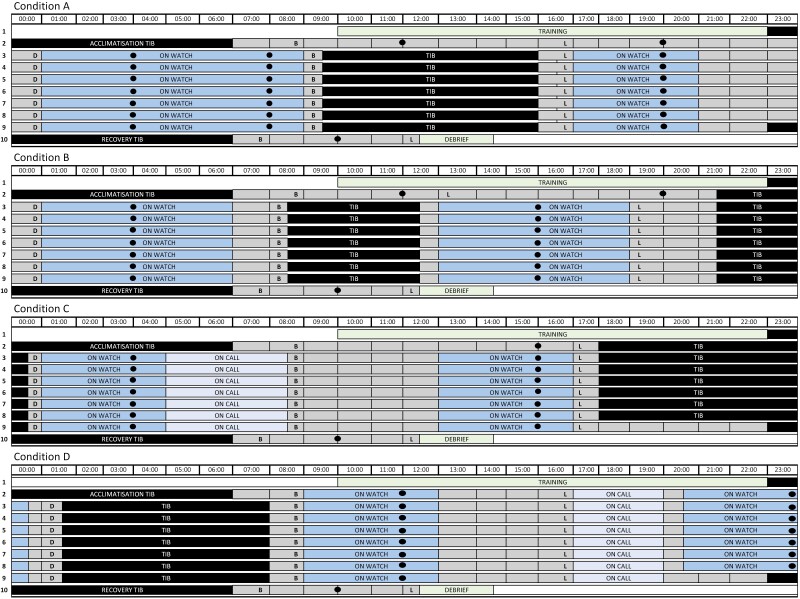
Study protocol. Study protocol for all conditions: condition A, daytime sleep of 8/8/4/4; condition B, day sleep split across two opportunities of 6/6; condition C, evening sleep of 4/4/4/4/4/4; condition D, nighttime sleep of 4/4/4/4/4/4. Abbreviations: B, breakfast; L, lunch; D, dinner; TIB, time in bed. Circles indicate ANAM testing sessions.

This live-in laboratory study consisted of eight random 10-day runs from December 2020 to February 2022. All study runs were conducted between late springs to early autumn at a moderate latitude (~35°C S), thus there was ~1 hour maximum difference in natural photoperiod between data collection periods. Consistent laboratory conditions of 22 ± 1°C and 100 lux at eye level during waking hours (0 lux during sleep periods) were maintained for the duration of the study runs. The laboratory was impermeable to natural light, and mobile phone use was prohibited as participants were restricted from knowledge of clock time during the study. Participants were randomized into one of the watch keeping schedule conditions, and up to four participants assigned to the same condition were studied each run. Thus, in each run, all participants were on the same schedule and permitted to socialize and recreate together during their downtime, as they would on a ship. After maintaining an actigraphy-verified (GENEActiv monitor, Activinsights 2022), consistently timed, nightly sleep duration of 7–9 hours for 1 week, participants arrived at the laboratory at ~10:00 on study day 1 (SD1) for training once toxicology was verified via urinalysis. All participants received a time in bed (TIB) of 8 hours on night 1 from 23:00 to 7:00 hours. Meals were controlled and isocaloric. Breakfast served at 08:30 on SD2 was common to all conditions, after which the schedules of each condition began to diverge. Reflecting the real-world nature of work, all schedules started with an on watch period (Figure 2). Participants maintained their respective simulated watch keeping schedules until realignment at 23:00 on SD9 for 8 hours recovery TIB. This permitted data collection over seven consecutive 24-hour cycles for each condition, all with a cumulatively equal daily 6.5 hours TIB. All participants were discharged from the laboratory at 14:00 on SD10 following debriefing.

### Watch keeping conditions

The experimental conditions are illustrated in Figure 2. Condition A was a watch section of a 2-section 8/8/4/4 schedule, with alternating 8 and 4 hours on watch periods, beginning at 01:00 and 17:00, respectively. In this section of the schedule, sleep was only permitted during the day from 9:30 to 16:00. Condition B was a watch section of the 2-section 6/6 schedule, with two 6 hours on watch periods beginning at 01:00 and 13:00. Condition B allowed for two sleep periods from 08:30 to 12:30 and 21:30 to 00:00, respectively. Condition C was a watch section of a 3-section 4/4/4/4/4/4 schedule, with two 4 hours on watch periods beginning at 01:00 and 13:00, respectively. Sleep was only permitted during the evening from 18:00 to 00:30. Condition D repeated the 3-section 4/4/4/4/4/4 schedule design as in condition C but represented a different section of this schedule with two 4 hours on watch periods beginning at 09:00 and 20:30, respectively. Sleep was only permitted at night from 01:30 to 08:00.

Conditions C and D both included a 4 hours “on call” period each day that were characterized by lower procedural tempo (i.e. the persistence of biological sampling and physiological measures) with no cognitive tasks. These times could be used to complete work that is not able to be done when “on watch.” At these times, personnel would not sleep but are also not on watch, thus these periods are distinguished from adjacent “on watch” periods on Figure 2. For conditions A, C and D, the TIB was restricted to a consolidated 6.5 hours during the 8 hours “off-watches,” as there are typically occupational barriers that restrict the duration of an “off-watch” period that can be devoted to sleep [21]. The daily 6.5 hours TIB was split into two periods of 4 and 2.5 hours in condition B, to mirror the splitting of sleep into two unequal durations per 24 hours, as has been reported to naturally occur in the 6/6 watch schedule [17].

### Higher order cognitive function testing

**Figure 3.**
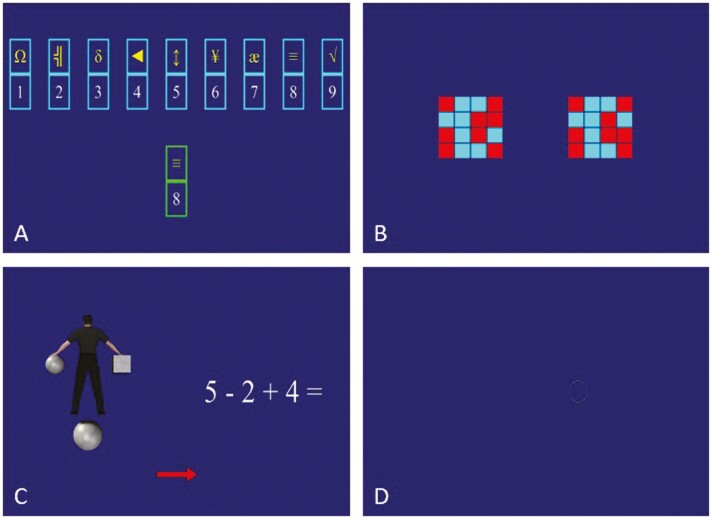
ANAM computer interface. Panels: (A) code substitution—learning (CDS); (B) matching to sample (M2S); (C) switching (SWT); (D) pursuit tracking (PUR).

The ANAM is a validated cognitive performance testing tool which has shown good psychometric properties and only modest practice effects, originally developed within the US Department of Defense for assessments in active-duty service members (ADSM) [22]. Five subtests from the ANAM library of 30+ subtest modules were included in the present study for their diverse representation of performance capacities. Two code substitution subtests were used: code substitution—learning (CDS) and code substitution—delayed (CDD), which collectively invokes attention, visual scanning, and perception, associative learning, processing speed, and recall [23]. In CDS, participants are continuously presented with nine unique pairings (key) of a digit (1–9) with a symbol (e.g. δ) and are trialed to indicate whether a single new pairing is correct based on the key (Figure 3A). Feedback is given immediately. In CDD, participants are trialed without the key, and no feedback is given. Matching to sample (M2S) examines working memory, visual short-term recognition memory, and spatial processing through presentation of a bicolored 4 × 4 grid pattern (sample) which must be subsequently identified from two bicolored 4 × 4 grid patterns (Figure 3B) [23]. Switching (SWT) combines two ANAM subtests (Manikin-Variation [MKN] and mathematical processing [MTH]) to collectively probe executive function, mental flexibility, computational skills, spatial rotation, and attention (Figure 3C) [23]. MTH trials participants by presenting simple three-integer equations to be solved (only +/− operators). MKN displays a man holding a cube in one hand and a ball in the other while either one is also displayed underneath the man (target). In each MKN trial, the man can face toward or away from the participant and be upright or upside down and participants must identify which hand is holding the target. SWT trials simultaneously display MTH and MKN on respective halves of the screen, along with a left–right arrow on the bottom indicating which of the two the participant is tasked with solving. The fifth subtest used was Pursuit Tracking (PUR) to assess visuomotor control by presenting participants with a bullseye within a moving circle and a mouse cursor which must be continually hand-guided to follow the bullseye as closely as possible (Figure 3D) [23].

Participants completed all administrations of the ANAM in a standardized fashion: independently in their respective rooms, under quiet conditions, free of distraction, on a desktop computer, during the “on watch” periods (Figure 2). Watch start timings were staggered across the conditions after baseline data collection on SD2. This, in conjunction with the timing structure of the respective schedules, resulted in unequal administrations of the ANAM across conditions for each study day and the study at large: 24 administrations for condition A, 17 for B and C, and 16 for D. The order and number of trials of the five ANAM subtests used was consistent for all ANAM administrations, with only CDD—which examines compounding recall interference—occurring more than once per session: e.g. CDS, PUR, CDD, SWT, CDD, M2S, and CDD. Each ANAM battery took ~25 minutes to complete as CDS, CDD, M2S, and SWT are self-paced (with 72, 108 [cumulatively], 20, and 64 trials, for each test, respectively) but PUR is a set time (2 minutes).

In accordance with previous research, three common metrics were derived from each CDS, CDD, M2S, and SWT: percent correct (PC; %), mean RT for correct responses (MRTC; ms), and throughput (TP; correct responses/min) [22]. Only one metric, percent in box (PIB), was derived from PUR. Therefore, an array totaling 13 ANAM metrics was analyzed in this study.

### Sleep

Sleep was recorded throughout this study with gold standard polysomnography (PSG) and scored by a certified sleep technician in accordance with standard criteria outlined by Rechtschaffen and Kales [24]. No PSG was recorded on SD5 to allow participants’ skin to recover from the wire-up procedure. The metrics of total sleep time (TST) and sleep efficiency (SE) were derived from these data and expressed in minutes and percentages, respectively.

### Data analysis

All analyses and visualizations were performed with the R (version 4.2.1) and RStudio (version 2022.07.2) software packages, utilizing the following libraries: *ggplot2, dplyr, tidyr, emmeans,* and *jtools.* Kruskal–Wallis tests and one-way ANOVAs were used to compare metrics between conditions at baseline and overall. Measures of effect size—partial eta-squared (η_p_2)—were calculated when differences between two or more conditions were detected for any metric, with thresholds set at 0.01, 0.06, and 0.14 for small, moderate, and large effect sizes, respectively [25]. For each of the thirteen metrics, conditions were ranked, when possible, based on statistically significant differences in performance to generate an overall performance ranking for the study. Top or bottom performance may be shared between two or more conditions for any given metric if their performance was indifferent to each other but significantly better (or worse) than at least one other condition. Monophasic sleep structure of conditions A, C, and D resulted in administrations of the ANAM at ~4 and ~16 hours since awakening (HSA) from SD3 through SD9 (Figure 2). This enabled Wilcoxon signed-rank analyses to be used to compare the changes in metrics from 4HSA to 16HSA within and between conditions A, C, and D, as an indicator of the stability of cognitive performance capacities over the waking day. Problems associated with multiple comparisons were addressed with the Holm adjustment method.

Differences in TST and SE between conditions were compared within the *emmeans* librarywith a Sidak correction as the post hoc, after TST was identified as a predictor from a linear mixed effect model run with participant ID as the random variable. An alpha value of 0.05 was designated as the threshold for statistical significance.

## Results

A total of 31 participants were enrolled in this study, however, five of these withdrew during the study. Data were included in the analysis for one participant who withdrew late in the study after having completed most testing sessions, for a final sample size of *N* = 27 (Figure 1; Table 1). Table 1 depicts participant demographics for the study. No significant differences in age or BMI were detected between groups.

**Table 1.** Participant Demographics

Condition	*n*	Female (%)	Age (years)	BMI (kg/m^2^)	TST (mins)^*^	SE (%)^*^
A	8	87.5	26.6 ± 6.2	23.7 ± 2.7	351.4 ± 62.4	86.4 ± 15.7
B	5	20	23.0 ± 2.7	24.3 ± 3.9	350.1 ± 51.8	84.2 ± 16.2
C	7	57.1	23.7 ± 4.9	24.3 ± 3.6	347.4 ± 71.9	85.4 ± 14.2
D	7	57.1	21.6 ± 5.6	22.7 ± 3.6	382.7 ± 34.3	93.8 ± 3.4
All	27	59.3	23.9 ± 5.3	23.7 ± 3.3	358.0 ± 58.6	87.1 ± 13.2

Abbreviations: BMI, body mass index; TST, total sleep time; SE, sleep efficiency. Age, BMI, TST, and SE are presented as mean ± standard deviation.

^*^Denotes significant differences between two or more groups.

### Performance testing

Significant differences between at least two conditions were yielded for every metric except for PC on CDS, none of which were detectable at baseline (Tables 2–4). Moderate effect sizes were observed for differences between conditions for at least one metric derived from visual scanning, learning, working memory, mental flexibility, but only a small effect size for visuomotor control metric of PIB. Tables 2–4 depict comparisons between conditions for the PC/PIB, MRTC, and TP metrics, respectively. Condition D exhibited the highest frequency of top performance (9 metrics), followed by condition A (6 metrics). Conditions B and C demonstrated the lowest frequency significantly outperforming at least one other condition (2 metrics; Table 5).

**Table 2.** Kruskal–Wallis Analysis of Percent Correct on the ANAM Subtests

	Condition	*n*	Mean	SD	Median	☐ ^2^	*p*	Condition comparisons	ϵ^2^
CDS	A	187	96.22	3.68	97.22	9.120	<.05^‡^	—	—
	B	83	96.69	2.25	97.22				
	C	94	97.21	2.50	97.22				
	D	111	97.28	2.68	98.61				
CDD	A	187	89.16	14.57	94.44	45.228	<.001	D > C^**^ B < A^***^, C^**^, D^***^	0.085
	B	83	78.56	17.53	83.33				
	C	94	87.99	12.12	90.28				
	D	112	93.13	7.62	94.44				
M2S	A	187	91.09	10.49	95.00	49.870	<.001	D > A, B, C^***^ A > C^***^	0.094
	B	84	90.48	8.09	92.50				
	C	94	84.56	14.14	90.00				
	D	111	95.05	7.37	100				
SWT	A	187	93.65	6.19	95.31	45.640	<.001	B < A^***^, C^*^, D^**^ C < A^**^, D^**^	0.085
	B	84	86.51	9.88	89.06				
	C	94	88.34	11.61	93.75				
	D	111	92.24	9.16	95.31				
PUR^†^	A	187	90.22	13.53	95.20	15.248	<.01	D > A^***^	0.024
	B	84	95.06	6.72	97.67				
	C	94	94.66	8.60	97.42				
	D	111	95.08	7.45	98.25				

Abbreviations: SD, standard deviation; CDS, code substitution—learning; CDD, code substitution—delayed; M2S, matching to sample; SWT, switching; PUR, pursuit tracking.

^†^PUR generates the outcome variable percent in box.

^‡^No significant differences were found between conditions for Dunn post hoc testing after p-value adjustment with Holm method. Angle brackets indicate significantly better performance (<, >). Level of significance is indicated (*p < .05, **p < .01, ***p < .001).

**Table 3.** Kruskal–Wallis Analysis of Mean RT for Correct Responses on the ANAM Subtests

	Condition	*n*	Mean	SD	Median	☐ ^2^	*p*	Condition comparisons	ϵ^2^
CDS	A	187	1232.04	283.36	1179.45	40.091	<.001	D > A^*^, B^*^, C^***^ C < A^***^, B^**^	0.074
	B	83	1219.49	180.94	1196.03				
	C	94	1415.46	339.65	1337.59				
	D	111	1158.74	256.11	1105.01				
CDD	A	187	1117.21	345.84	1062.89	32.278	<.001	B > A^**^, C^***^, D^*^ C < A^**^, D^**^	0.058
	B	83	950.23	299.16	957.76				
	C	94	1257.69	369.91	1157.81				
	D	112	1080.47	226.27	1027.71				
M2S	A	187	1338.15	314.60	1334.35	29.945	<.001	A > B^***^, C^***^ D > B^**^, C^**^	0.054
	B	84	1523.84	354.93	1502.90				
	C	94	1705.16	659.19	1451.76				
	D	111	1376.24	325.01	1338.60				
SWT	A	187	1862.59	454.55	1774.17	28.159	<.001	B < A^***^, C^***^, D^*^ C > D^*^	0.050
	B	84	2169.10	536.17	2141.20				
	C	94	1796.00	594.54	1625.12				
	D	111	1999.42	639.25	1819.94				

Abbreviations: SD, standard deviation; CDS, code substitution—learning; CDD, code substitution—delayed; M2S, matching to sample; SWT, switching. Angle brackets indicate significantly better performance (<, >). Level of significance is indicated (*p < .05, **p < .01, ***p < .001).

**Table 4.** Kruskal–Wallis Analysis of Throughput on the ANAM Subtests

	Condition	*n*	Mean	SD	Median	☐ ^2^	*p*	Condition comparisons	ϵ^2^
CDS	A	187	49.02	10.37	49.57	39.583	<.001	D > A^*^, B^*^, C^***^ C < B^**^, A^***^	0.073
	B	83	48.38	6.38	48.60				
	C	94	43.30	9.19	43.75				
	D	111	52.46	10.33	52.65				
CDD	A	187	50.98	14.36	51.46	18.522	<.001	C < A^**^, B^*^, D^***^	0.031
	B	83	52.60	16.72	49.03				
	C	94	44.98	14.08	44.91				
	D	112	53.18	11.92	54.64				
M2S	A	187	42.27	11.95	41.34	41.795	<.001	A > B^***^, C^***^ D > B^***^, C^***^	0.078
	B	84	36.83	10.48	35.15				
	C	94	33.79	14.73	31.31				
	D	111	42.85	10.00	42.28				
SWT	A	187	31.47	7.20	31.63	31.654	<.001	B < A^***^, C^***^, D^**^	0.057
	B	84	26.20	7.10	24.45				
	C	94	32.78	10.68	33.86				
	D	111	30.26	7.87	30.97				

Abbreviations: SD, standard deviation; CDS, code substitution—learning; CDD, code substitution—delayed; M2S, matching to sample; SWT, switching. Angle brackets indicate significantly better performance (<, >). Level of significance is indicated (*p < .05, **p < .01, ***p < .001).

**Table 5.** ANAM Performance Ranking

Subtest (cognitive domain)	Top performance	Bottom performance
*CDS (visual scanning)*		
PC	—	—
MRTC	D	C
TP	D	C
*CDD (learning)*		
PC	A, D	B
MRTC	B	C
TP	A, B, D	C
*M2S (working memory)*		
PC	D	—
MRTC	A, D	B, C
TP	A, D	B, C
*SWT (mental flexibility)*		
PC	A, D	B
MRTC	C	B
TP	A, C, D	B
*PUR (visuomotor control)*		
PIB	—	—

Abbreviations: CDS, Code Substitution—Learning; PC, Percent Correct; MRTC, Mean Reaction Time for Correct Responses; TP, throughput; CDD, code substitution—delayed; M2S, matching to sample; SWT, switching; PUR, pursuit tracking; PIB, percent in box. Significant differences between conditions were not always sufficient to elucidate a top and/or bottom performing condition.

**Figure 4.**
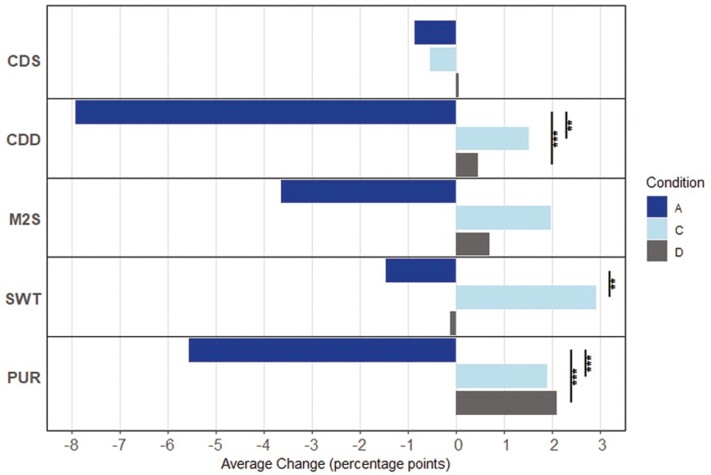
Conditions A, C and D average change in PC/PIB from 4HSA to 16HSA. Abbreviations: PC, percent correct; PIB, percent in box; CDS, code substitution—learning; CDD, code substitution—delayed; M2S, matching to sample; SWT, switching; PUR, pursuit tracking. Level of significance is indicated (**p < .01, ***, *p* < .001).

**Figure 5.**
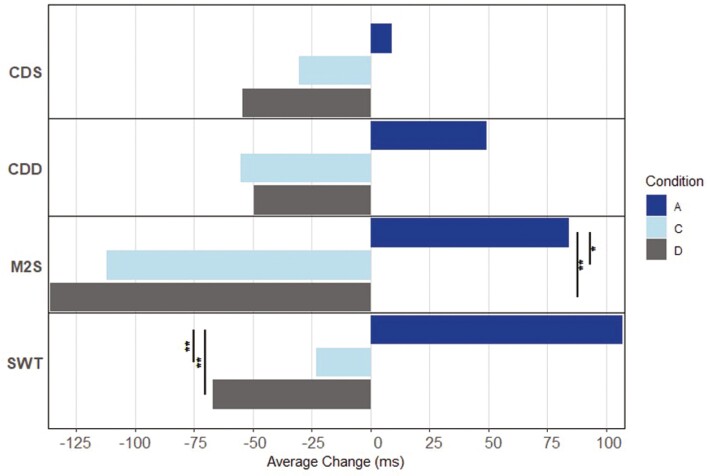
Conditions A, C, and D average change in MRTC from 4HSA to 16HSA. Abbreviations: MRTC, mean reaction time for correct responses; HSA, hours since awakening; CDS, code substitution—learning; CDD, code substitution—delayed; M2S, matching to sample; SWT, switching; ms, milliseconds. Level of significance is indicated (*p < .05, **p < .01).

**Figure 6.**
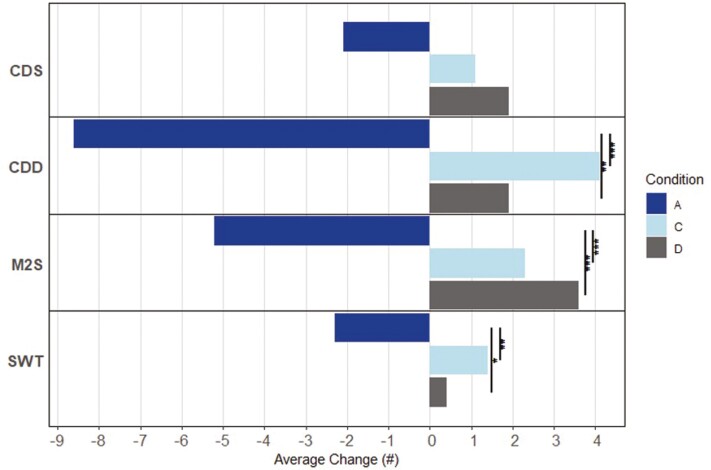
Conditions A, C, and D average change in TP from 4HSA to 16HSA. Abbreviations: TP, throughput; HSA, hours since awakening; CDS, code substitution—Learning; CDD, code substitution—delayed; M2S, matching to sample; SWT, switching. Level of significance is indicated (*p < .05, **p < .01, ***p < .001).

Within-condition, significant changes in performance from 4HSA to 16HSA from SD3—SD9 were only detected for condition A, such that performance on average, was degraded at 16HSA compared to 4HSA on 6 of the 13 metrics. Conversely, nonsignificant trends of improvement at 16HSA compared to 4HSA were observed for several metrics for conditions C and D. Between conditions, the magnitude of this bidirectionality underscored significantly lower stability over the waking day for condition A compared to conditions C and D for seven metrics (Figures 4–6).

### Sleep

On average, as would be expected, condition D exhibited the highest TST of any condition, which was significantly higher than condition B, but not that of conditions A or C (Table 1). Similarly, condition D exhibited the highest SE of any condition, which was significantly greater than conditions B and C (Table 1).

## Discussion

The current study examined visual scanning, learning, working memory, mental flexibility, and visuomotor control in four simulated watch keeping schedule sections. Previous studies of cognitive performance during watch keeping have not often compared between schedules. In this laboratory study, condition D (nighttime sleep of 4/4/4/4/4/4) received the highest ranking in higher order cognition, followed by condition A (daytime sleep of 8/8/4/4). Condition B (day sleep split across two opportunities of 6/6) received the lowest ranking based on performance, closely followed by condition C (evening sleep of 4/4/4/4/4/4). This ranking was consistent with performance modeling by Paul et al., and generally with the larger body of work in the laboratory and the field, which suggests that performance is better supported during shorter watches [9, 10, 15, 18].

The generally recommended daily sleep duration for maintaining optimum performance levels is 7–9 hours for adults. Work by Belenky et al. has shown that only mild sleep restriction over several days (7 hours TIB for 7 days) can result in cumulatively degraded vigilance [26]. Given the uniform allocation of only 6.5 hours daily TIB in the present study, there was sleep restriction in all conditions. Accordingly, all conditions demonstrated varied, but overall negative effects on different domains of cognitive performance. Conditions A (sleep during day), B (split sleep periods), and C (sleep in evening) were lowest in performance in visual scanning, learning, working memory, mental flexibility, and visuomotor control. Condition D, with a sleep period at night, was generally ranked highest for most tasks. Particularly, condition B, which had sleep split across two periods, exhibited the worst performance for SWT—an important component of multitasking. Poor performance has been found previously as a consequence of this schedule due to reduced sleep time and the fact there are two daily sleep inertia periods inherent to biphasic sleep [27, 28]. For example, Dahlgren et al. reported worse sleepiness and fatigue in the night work compared to day work watch of the 6/6 [29].

Despite the uniform allocation of daily TIB (6.5 hours), there were small differences in sleep (TST and SE) between conditions that are likely due different timing of the sleep periods and the circadian propensity for sleep at their respective times. For example, participants within condition D which had a sleep period at night, had slightly longer TST. The differences in cognitive performance between conditions could also be due to other factors in addition to the small differences in sleep, including the timing of the sleep period and the timing of performance testing during the watch period. For instance, condition D, the top performer, which was the only condition to have a consolidated nighttime sleep period, also thereby had no performance testing around the circadian nadir, when performance is known to deteriorate. Conversely, condition A contained the longest duration of nightwork of any condition and included one additional test per 24 hours compared to the other conditions. These factors are also likely to have underscored differences between conditions A, C, and D in the stability of performance during the waking period, even though they all had consolidated rather than split sleep periods. The differences were likely largely an artifact of the circadian influence at the timing of the tests (i.e. working through the biological night when performance is impaired relative to the biological day). While some of the conditions were less problematic for cognitive performance than others, performance was affected in each condition. This suggests that when around-the-clock work is required in conjunction with the other challenges associated with watch keeping, cognition is vulnerable.

Strategic, principles-based schedule engineering is an important component of mitigating fatigue, a major cause of performance impairment during watch keeping [30, 31]. A few guiding principles have emerged for shaping viable watch keeping schedules, including: 24-hour structure, consistent timing of daily activities, adequate spacing between “on-watch” shifts (>3 hours), and longer-end “off-watch” periods (8–10 hours) [12, 28, 31, 32]. However, the implementation of these principles can be at odds with the culture surrounding watch keeping. Thus, the investigation and comparison of schedules that can meet unique operational requirements and conditions (e.g. reduced manning) whilst prioritizing these principles should continue to be explored with the aid of broad, validated performance measures. Knowledge gained from such studies is best applied in concert with other fatigue-mitigation tactics. Studies have suggested fostering mindfulness, sleep hygiene education, and personalized lighting hardware may all have utility in managing fatigue and conserving performance during watch keeping [33–35].

There are a few limitations to be considered with the present study. As the aim of this study was to compare sections of the 8/8/4/4, 6/6, and 4/4/4/4/4/4 schedules with most circadian misalignment in sleep timing, only those select sections of these schedules were investigated and the whole schedule was not examined. The rankings generated therefore may not reflect the cognitive performance of the whole watch keeping schedule. Watch keeping studies that examine performance in ADSM watch keepers in the laboratory, as was done by Miller et al. have the advantage of more closely simulating watch keeping conditions [15]. Additionally, as all participants were intermediate chronotypes, we were unable to examine if chronotype influenced the adaptability of these schedule sections. The female predominance of our sample helps to fill an important gap in knowledge around how these schedules impact female populations. Many previous studies have a much higher male to female ratio, traditionally consistent with the military population. Many Navies around the world are aiming to include more women. Notwithstanding, our small sample size did not enable us to control for gender in our performance analyses, which prior studies have shown can yield differences in response to sleep loss [36]. Lastly, Banks et al. highlighted the impact that sleep history can have on performance several weeks prior to assessment [37]. As prestudy sleep was controlled for only one week in the present study, longer term sleep history could have impacted our findings.

This study found that higher order cognitive function was ranked worse for the 6/6 schedule section where sleep was split across two periods during the biological day and work was during the biological night. However, the stability of higher order cognitive function was poorest in the 8/8/4/4 section where sleep was consolidated, but during the biological day and there was a longer work period at night. These findings highlight the variability in cognitive capacities during different watch keeping schedules and how timing of sleep and work influence performance. Further work is needed to look at these schedules in their entirety, but results here suggest that a consolidated sleep, as in a 3-watch schedule, has benefits for higher order cognitive function.

## Data Availability

The study was funded by Defence Science and Technology Group (Australia). Data restrictions apply. Data may be available with the permission of Defence Science and Technology Group (Australia).

## Notes

This paper is part of the Sleep and Circadian Rhythms: Management of Fatigue in Occupational Settings Collection.
